# Assessment of Liver Function Using Pharmacokinetic Parameters of Gd-EOB-DTPA: Experimental Study in Rat Hepatectomy Model

**DOI:** 10.1155/2018/6321316

**Published:** 2018-03-11

**Authors:** Myung-Won You, Hyoung Jung Kim, Hyeong-Seok Lim, So Yeon Kim, Jae Ho Byun, Kyung Won Kim, Dae Wook Hwang, Young-Joo Lee

**Affiliations:** ^1^Department of Radiology and the Research Institute of Radiology, University of Ulsan College of Medicine, Asan Medical Center, Seoul, Republic of Korea; ^2^Department of Radiology, Eulji Hospital, Eulji University School of Medicine, Seoul, Republic of Korea; ^3^Department of Clinical Pharmacology and Therapeutics, University of Ulsan College of Medicine, Asan Medical Center, Seoul, Republic of Korea; ^4^Department of Surgery, University of Ulsan College of Medicine, Asan Medical Center, Seoul, Republic of Korea

## Abstract

**Objectives:**

To determine whether the pharmacokinetic parameters of Gd-EOB-DTPA can identify the difference in liver function in a rat hepatectomy model.

**Methods:**

A total of 56 eight-week-old male Sprague-Dawley rats were divided into the following groups: control group without hepatectomy (*n* = 16), 70% hepatectomy group (*n* = 14), and 90% hepatectomy group (*n* = 26). On postoperative day 2, Gd-EOB-DTPA (0.1 mmol/kg) was injected intravenously and serial blood samples were obtained. Pharmacokinetic analysis was performed using a noncompartmental method. Statistical analysis was performed using one-way analysis of variance and post hoc pairwise group comparisons.

**Results:**

After excluding 6 rats that died unexpectedly, blood samples were obtained from 16, 14, and 20 rats in the control group, 70% hepatectomy group, and 90% hepatectomy group. There was a significant increase in area under the concentration-time curve from time zero to the time of the last measurable concentration between the 70% and 90% hepatectomy group (*P* < 0.001). The volume of distribution at steady state was significantly decreased between the control and 70% hepatectomy group (*P* < 0.001). The clearance was significantly different in all pairwise group comparisons (*P* < 0.001).

**Conclusions:**

The vascular clearance of Gd-EOB-DTPA can identify the difference in liver function in a rat hepatectomy model.

## 1. Introduction

Assessment of liver function is important to determine the prognosis in patients with chronic liver disease, to establish the optimal timing for transplantation or transjugular intrahepatic portosystemic shunt insertion, and to minimize the risk of hepatic failure following major hepatic resection [1, 2]. In daily clinical practice, serum bilirubin level, serum albumin level, and prothrombin time are methods that are simple and commonly used to assess hepatic function. However, these qualitative liver function tests cannot exactly reflect liver function, because a single measurement of an activity or the concentration of a substance in the blood does not provide data about the volume of distribution, amount of production, or amount of elimination [2]. Thus, when more precise assessments of liver function are required, for example, before major hepatectomy, quantitative liver function tests, such as indocyanine green (ICG), are favored [2].

Hepatic uptake and biliary excretion of ICG involve an organic anion transporter (OAT) system which is increasingly recognized as a major route for the transport of anionic xenobiotics and endogenous substances into the bile [3]. The expression of OATs in the liver varies in acute and chronic liver disease and accordingly it is considered to be an important indicator of liver function [4, 5]. Interestingly, Gd-EOB-DTPA, which is a widely used contrast agent for liver MR imaging, uses the same OAT as ICG [6–8]. Recent studies used Gd-EOB-DTPA-enhanced liver MR imaging to evaluate liver functions [9–17]. These studies mainly focused on enhancement of the hepatic parenchyma on Gd-EOB-DTPA-enhanced liver MR imaging. However, hepatic parenchymal enhancement on Gd-EOB-DTPA-enhanced liver MR is associated with decreased serum concentrations of the contrast agent. Therefore, measurements of serum Gd-EOB-DTPA concentrations can represent a more direct method to assess hepatic function than evaluations of hepatic parenchymal enhancement on liver MR imaging.

The multiple and various functions of the liver preclude an easy single reference standard for assessments of liver function [18]. In the rat hepatectomy model, defined combinations of liver lobes are removed, which are classified based on the approximate relative resected liver mass as 30%, 50%, 70%, and 90% hepatectomy [19, 20]. A reference standard of liver function based on rat hepatectomy model may represent overall liver functions and hepatectomy with increasing lobe resections is a convenient model to assess liver functions. The purpose of this study was to determine whether pharmacokinetic parameters of Gd-EOB-DTPA could identify the difference in liver function in a rat hepatectomy model.

## 2. Materials and Methods

This study was reviewed and approved by the Institutional Animal Care and Use Committee (IACUC) ^*∗*^BLINDED^*∗*^ Institute for Life Sciences, ^*∗*^BLINDED^*∗*^ Medical Center. The committee abides by the institute of Laboratory Animal Resources (ILAR) guide.

### 2.1. Animals

A total of 56 eight-week-old male Sprague-Dawley (SD) rats (Orientbio Inc., Korea) were used. Several major surgical organizations have established guidelines that recommend that at least 20% of a normal liver with intact vascular and biliary tree should remain for the surgical resection of liver tumors [21]. Therefore, we divided rats into the following groups: control group without hepatectomy (*n* = 16), 70% hepatectomy group (*n* = 14), and 90% hepatectomy group (*n* = 26). The 70% hepatectomy group was designed to represent hepatectomy with abnormal but sufficient liver function for survival. The 90% hepatectomy group was designed to represent hepatectomy with marginal liver function for survival.

### 2.2. Rat Hepatectomy

Each rat was weighed prior to surgery. Anesthesia and rat hepatectomy were performed by an experienced veterinarian (S.H.H., who had 8 years of experience in animal surgery). For anesthesia, an isoflurane vaporizer (RC2, Vetequip, USA) with an isoflurane concentration of 1%–4% and an oxygen flow rate of 0.5 L/min was used. Removal by crude ligation of the wide base of the median lobe and right superior lobe may cause constriction of the vena cava and subsequent functional impairment of the remnant liver [19]. Therefore, we used the clamping and piercing suture technique proposed by Madrahimov et al. [19]. After placing a Mosquito clamp around the base of the each lobe, the liver tissue was dissected just above the clamp. Piercing sutures that penetrated the entire parenchyma were placed below the clamp. Immediately after the operation, animals received 1.5 mL 10% glucose and 1.5 mL normal saline subcutaneously, along with intramuscular 0.2 mL Gentamycin (Choongwae, Korea) and 0.2 mL Diclofenac Sodium (Samjin, Korea). Animals had free access to water and feed.

### 2.3. Blood Sampling

Blood sampling was performed on postoperative day (POD) 2. Each rat was weighed prior to blood sampling. Femoral artery cannulation was performed for blood sampling. Gd-EOB-DTPA (Primovist; Bayer HealthCare, Berlin, Germany) (0.1 mmol gadolinium per kg body weight, 0.4 mL/kg) was used for pharmacokinetic analyses. The Gd-EOB-DTPA was diluted 15-fold using normal saline and injected into the tail vein. Blood samples of 120 *μ*L were obtained 1, 3, 5, 10, 20, 30, 60, and 90 min after injecting Gd-EOB-DTPA. For each 120 *μ*L of blood, 50 *μ*L was used for gadolinium measurement and 70 *μ*L was reserved.

### 2.4. Measurement of Liver Weight

All animals were sacrificed using a CO_2_ chamber after the completion of blood sampling. In each group, the wet liver weight was measured after explantation. The total wet liver weight was measured after removing the inferior vena cava and portal vein. To determine the absolute weight of each liver lobe, the weight of each liver lobe was also measured after dissecting each lobe with the same technique for rat hepatectomy. The relative weight of each lobe was calculated using the following formula: relative weight of lobe (%) = weight of lobe (g)/total liver weight (g).

### 2.5. Gadolinium Measurements and Pharmacokinetic Analysis

Blood samples were assayed for gadolinium using an inductively coupled plasma-mass spectrometry (ICP-MS). Blood samples were centrifuged to collect all material at the bottom of the caps. We added 50 *μ*L internal standard (100 nM Terbium-nitrate). The mixture was dried at 90°C. Then, 20 *μ*l 30% hydrogen peroxide and 50 *μ*L concentrated nitric acid were added and the mixture was heated to 120°C for about 30 min at increased pressure. After cooling, the volume was made up to 1 mL with water. The solution was further diluted depending on the expected gadolinium concentration in the samples. Final dilutions were injected to the ICP-MS to obtain the concentration measurements. The ICP-MS (Agilent 7900) was calibrated using dilutions of commercial, certified standards (Merck) with 0, 1, 10, and 100 nmol Gd/L and 10 nM Tb. All sample measurements were made within the calibrated range. Blank digestions (empty caps) were also included in the procedure and resulted in Gd concentrations well below the limit of quantification. Using our new ICP-MS, the limit of quantification was about 0.1 nmol Gd/L. The upper limit of the linear measurement range was about 20 *μ*mol Gd/L. Serial serum concentration-time profiles of gadolinium for each rat were analyzed using a noncompartmental method with the WinNonlin 6.3 (Pharsight Corporation, Mountain View, CA, USA). All analyses were made based on actual times of sampling. Pharmacokinetic analysis was performed using a noncompartmental method [22, 23]. The individual area under the concentration curve (AUC) from time zero to time of last measurable concentration was indicated as AUC_last_ and AUC*∗*time versus time curve from time zero to time of last measurable concentration was indicated as AUMC_last_. These AUC_last_, AUMC_last_ and time product were estimated by linear trapezoidal summation in the ascending period and by log/linear trapezoidal summation in the descending period. The AUC from time zero extrapolated to infinite time (AUC_inf_) and the AUMC from time zero extrapolated to infinite time (AUMC_inf_) were calculated as the sum of the AUC_last_ and *C*_last_/*λ*_z_  (AUC_extrapolated_) and as the sum of AUMC_last_ and *C*_last_*∗*time/*λ*^*∗*^_z_  (AUMC_extrapolated_), respectively, in which *C*_last_ corresponds to the last predicted concentration. *λ*_z_ and *λ*^*∗*^_z_ are the rate constant of the terminal phase calculated by linear regression of the slope of the terminal portion of the log-transformed serum concentration versus time curve and serum concentration*∗*time versus time curve, respectively. Clearance (CL) was computed as the dose/AUC_inf_. *V*_z_ was volume of distribution during terminal phase (CL/*λ*_z_, terminal phase refers to post distribution or elimination phase), and *V*_ss_ was volume of distribution at steady state (dose*∗*(AUMC/AUC^2^), steady state refers to distribution phase at free concentration in plasma being equal to the free concentration in the tissue). The mean residence time (MRT) was calculated as AUMC/AUC. The terminal elimination half-life (*t*_1/2*β*_) was calculated for each subject as ln⁡(2)/*λ*_z_, and the effective half-life (*t*_1/2,eff_) was calculated as 0.693*∗*MRT.

### 2.6. Statistical Analysis

Data were expressed as means ± standard deviation. For comparisons of the three groups,* *continuous variables were compared using one-way analysis of variance. A *P* value of less than 0.050 was considered to indicate a significant difference. When data indicated the presence of significant difference between the three groups, post hoc pairwise group comparisons were made using Student's *t* test with the Bonferroni correction. Spearman's correlation test was performed and a scatter plot was used to show the correlation between CL and three groups. All analyses were performed using SPSS 21 (SPSS Inc., Chicago, IL, USA).

## 3. Results

### 3.1. Subjects

The control group consisted of 16 SD male rats and blood sampling was possible in all 16 animals. The 70% hepatectomy group consisted of 14 SD male rats and blood sampling was possible in all 14 animals. The 90% hepatectomy group consisted of 26 SD male rats and blood sampling was possible in 20 animals. Two rats died during hepatectomy as a consequence of hemorrhage and four rats died during the postoperative period. Finally, overall 50 rats were included in the pharmacokinetic analysis.

### 3.2. Liver Weight

Pre- and postoperative body weights and the explanted liver weights in each group are summarized in Table 1. In the control group (*n* = 16), the weight of total liver was 12.87 ± 1.70 g, left lateral lobe was 3.98 ± 0.58 g, median lobe was 4.26 ± 0.68 g, right lobe was 2.23 ± 0.37 g, caudate lobe was 0.82 ± 0.19 g, and paracaval portion was 1.42 ± 0.49 g. The relative weights were as follows: left lateral lobe, 30.9%; median lobe, 33.1%; right lobe, 17.4%; caudate, 6.4%; and paracaval portion, 11.0%. The sum of the relative weight of the left lateral and median lobe was 64.1%. The sum of the relative weight of the left lateral, median, and right lobe was 81.4%. In the 70% hepatectomy group (*n* = 14), the weight of the remnant liver at POD 2 was 8.49 ± 0.90 g. In the 90% hepatectomy group (*n* = 20), the weight of the remnant liver at POD 2 was 4.82 ± 0.70 g.

### 3.3. Pharmacokinetics of Gd-EOB-DTPA

**Figure 1:**
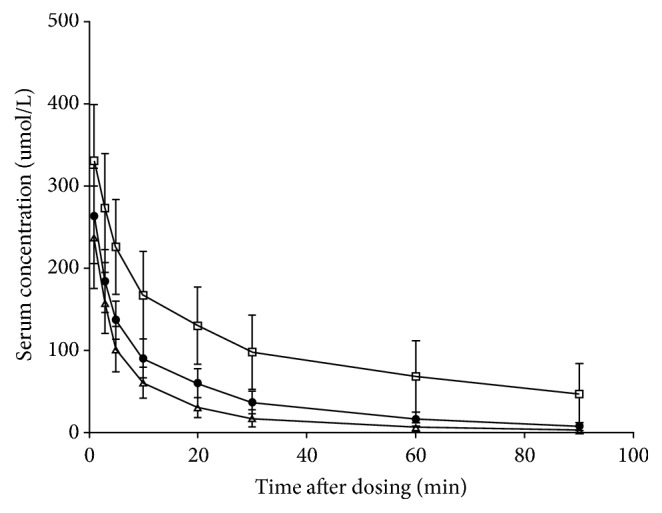
Mean serum concentration-time profiles of Gd-EOB-DTPA in the control group (△), 70% hepatectomy group (●), and 90% hepatectomy group (□). The error bars indicate the standard deviation.

**Figure 2:**
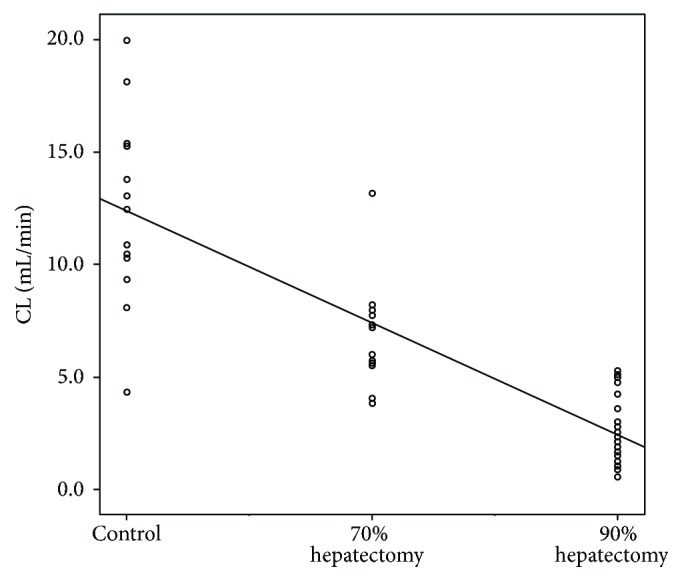
Clearance (CL) of Gd-EOB-DTPA as a function of the control group, 70% hepatectomy group, and 90% hepatectomy group showing significant decrease of parameter values with increasing extent of rat hepatectomy (*r* = −0.876, *P* < 0.001).

A summary of the Gd-EOB-DTPA serum concentration-time profiles for the three groups is shown in Figure 1. Pharmacokinetic parameters obtained from these data are summarized in Table 2. Results of overall and pairwise group comparisons of the three groups are summarized in Table 3. Serum Gd-EOB-DTPA concentrations declined rapidly during the first 10 min and then slowly declined until 90 min after injection in the control group (Figure 1), indicating nonlinear pharmacokinetics. This initial rapid reduction in serum Gd-EOB-DTPA concentrations was similar in the 70% and 90% hepatectomy group. In pairwise group comparisons, all pharmacokinetics parameters showed significant difference between the 70% and 90% hepatectomy group and between control and 90% hepatectomy group. Only CL, *V*_ss_, and *V*_z_ showed significant decrease between the control and 70% hepatectomy group. The CL, *V*_ss_, and *V*_z_ were significantly different in all pairwise group comparisons. Scatter plots showing a correlation of CL for the three groups had a significant negative correlation (*r* = –0.876, *P* < 0.001) (Figure 2).

## 4. Discussion

Our present study shows that measurements of serum Gd-EOB-DTPA concentrations can identify the difference in liver function in a rat hepatectomy model. Among pharmacokinetic parameters, there was a significant increase in the AUC parameters between 70% and 90% hepatectomy group. *V*_ss_ was significantly decreased between the control and 70% hepatectomy group. The CL was significantly different in all pairwise group comparisons.

The volume of distribution parameters (*V*_ss_ and *V*_z_) revealed a significant decrease between the control and 70% hepatectomy group, and they showed a marginally significant difference between 70% and 90% hepatectomy group. *V*_ss_ and *V*_z_ represented the apparent volume of distribution of Gd-EOB-DTPA during steady state and terminal phase. The difference in liver volume was greater between the control and 70% hepatectomy group than those between the 70% and 90% hepatectomy group, which may explain the difference in *V*_ss_ and *V*_z_ among the three groups. Interestingly, the difference in explanted liver weight between the control and 70% hepatectomy group was similar to that between the 70% and 90% hepatectomy group. The explanted liver weight of the 70% or 90% hepatectomy group was not the normal liver weight but the regenerated liver weight. In our study, the regeneration rate of 70% and 90% hepatectomy group was about 65% and 37% at POD 2, similar to those of 70% and 90% hepatectomy group (70% and 40%) in the previous literatures [24, 25]. In daily clinical practice, the calculated regenerated liver volume following portal vein embolization does not always correlate with liver function [18]. Therefore, differences in *V*_ss_ and *V*_z_ between the three groups might represent the differences in liver volume caused by rat hepatectomy rather than the absolute weight of the regenerated liver at POD 2.

On the other hand, the AUC and its related parameters were significantly increased between the 70% and 90% hepatectomy group but not between the control and 70% hepatectomy group. The AUC_last_ and AUC_inf_ are important pharmacokinetic parameters for noncompartmental analysis as they are frequently used to determine other pharmacokinetic parameters. These results indicate that a critical point in the pharmacokinetics of Gd-EOB-DTPA may present between the 70% and 90% hepatectomy. That is, liver with 30% remnant volume may be sufficient for survival while liver with 10% remnant volume may be marginal for survival. Our rat hepatectomy model is very useful to verify such findings.

The CL can be defined as a measurement of the volume of plasma that is completely cleared from a substance per unit of time [26]. CL is one of the primary, independent pharmacokinetic parameters. As the total body CL of Gd-EOB-DTPA will be equal to the hepatic CL plus renal CL, a significant difference in total body CL may result from difference in liver function in a rat hepatectomy models.

Our present study demonstrated that direct measurements of the serum Gd-EOB-DTPA concentrations may represent a novel quantitative liver function test. Among various pharmacokinetic variables, CL showed significant differences in all pairwise group comparisons. However, clinical studies with a large patient cohort will be required to determine whether CL of Gd-EOB-DTPA could represent a new quantitative liver function test. If blood sampling can be performed during Gd-EOB-DTPA-enhanced liver MR imaging, prerequisite data prior to liver surgery, such as liver function, volume, and anatomy, may be obtained simultaneously.

There were several limitations to our present study. First, we did not assess the urinary excretion of Gd-EOB-DTPA. Urinary bladder catheterization in male SD rat was impossible because of the long and tortuous course of the urethra. Second, the dosage of Gd-EOB-DPTA used in our study (0.1 mmol/kg) is 4-fold greater than the dosage used in human (0.025 mmol/kg). We used a high dose of Gd-EOB-DTPA to compensate much faster metabolic rate of SD rat than human [27]. A previous study also showed that serum concentration-time profile for a dose of 0.05 mmol/kg was similar to that of 0.5 mmol/kg in an experiment that used rats [28]. Third, the number of SD rats in each group was different. Initially, the expected number of SD rats without unwanted events in each group was 12. However, exclusion of the SD rats with unwanted events, such as hemorrhage, inflammation, or various injection sites other than the tail vein, may cause the selection bias. So, we determined to include all SD rats in which successful blood sampling was achieved to prevent the bias introduced by the arbitrary selection of cases.

In conclusion, measurements of serum Gd-EOB-DTPA concentrations can identify the significant difference in liver function in a rat hepatectomy model. Among the various pharmacokinetic parameters, CL of Gd-EOB-DTPA would be the most appropriate parameter to identify the difference in liver function and might have utility as a novel quantitative liver function test.

## Figures and Tables

**Table 1:** The body weight and explanted liver weight in each study group of SD rats.

	Control (*n* = 16)	70% hepatectomy (*n* = 14)	90% hepatectomy (*n* = 20)
Preoperative body weight (g)	303.69 ± 24.52	287.14 ± 13.82	296.00 ± 23.86
Postoperative body weight	NA	268.21 ± 15.36	262.55 ± 21.23
Explanted liver weight (g)	12.87 ± 1.70	8.49 ± 0.90	4.82 ± 0.70

NA: not available.

**Table 2:** The summary of pharmacokinetic parameters of Gd-EOB-DTPA.

	Control (*n* = 16)	70% hepatectomy (*n* = 14)	90% hepatectomy (*n* = 20)
AUC_last_ (*μ*mol·min/L)	2501.0 ± 922.6	3956.6 ± 1067.2	9234.5 ± 3891.5
AUC_inf_ (*μ*mol·min/L)	2659.6 ± 1099.2	4292.3 ± 1281.8	14199.8 ± 10949.0
AUC_extrapolated_ (%)	5.1 ± 3.2	7.1 ± 3.3	24.7 ± 15.2
AUC_inf_/dose (*μ*mol·min/L/*μ*mol)	90.1 ± 43.1	160.6 ± 49.0	545.0 ± 417.5
*C*_max_ (*μ*mol/L)	238.1 ± 62.4	264.1 ± 58.2	334.9 ± 65.6
*C*_max_/Dose (*μ*mol/L/*μ*mol)	8.0 ± 2.6	9.9 ± 2.3	12.9 ± 2.9
CL (ml/min)	12.7 ± 4.1	6.8 ± 2.3	2.7 ± 1.5
*V*_ss_ (mL)	269.7 ± 70.1	189.1 ± 36.0	142.1 ± 39.3
*V*_z_ (mL)	487.7 ± 157.0	254.9 ± 62.1	160.6 ± 55.2
*t*_1/2*β*_ (min)	27.3 ± 5.9	27.0 ± 5.3	52.6 ± 30.2
*t*_1/2,eff_ (min)	15.6 ± 4.5	20.3 ± 4.5	48.1 ± 30.1

Data are presented as means ± standard deviation. AUC_last_: area under the serum concentration-time curve from time zero to time of last measurable concentration; AUC_inf_: AUC from time zero to time extrapolated to infinite time; AUC_extrapolated_ (%): AUC from time of last measurable concentration extrapolated time to infinite time/AUC_inf_; *C*_max_: measured peak plasma concentration; CL: clearance; *V*_ss_: steady-state volume of distribution; *V*_z_: terminal phase volume of distribution; *t*_1/2*β*_: terminal half-life; *t*_1/2,eff_: effective half-life.

**Table 3:** The effects of hepatic resection on pharmacokinetic parameters of Gd-EOB-DTPA.

	Overall comparison^*∗*^	Pairwise group comparison^†^		
		Control versus 70% H	70% H versus 90% H	Control versus 90% H
AUC_last_ (*μ*mol·min/L)	<.001	.394	<.001	<.001
AUC_inf_ (*μ*mol·min/L)	.019	1.000	.001	<.001
AUC_extrapolated_ (%)	<.001	1.000	<.001	<.001
AUC_inf_/Dose (*μ*mol·min/L/*μ*mol)	<.001	1.000	<.001	<.001
*C*_max_ (*μ*mol/L)	<.001	.785	.007	<.001
*C*_max_/Dose (*μ*mol/L/*μ*mol)	<.001	.172	.007	<.001
CL (ml/min)	<.001	<.001	<.001	<.001
*V*_ss_ (mL)	<.001	<.001	.031	<.001
*V*_z_ (mL)	<.001	<.001	.030	<.001
*t*_1/2*β*_ (min)	<.001	1.000	.002	.001
*t*_1/2,eff_ (min)	<.001	1.000	<.001	<.001

^*∗*^One-way analysis of variance; ^†^post hoc pairwise group comparisons by using Student's *t* test with Bonferroni correction; H: hepatectomy.

## Abbreviations

SD: Sprague-Dawley
AUC: Area under the concentration-time curve
*C*_max_: Measured peak plasma concentration
CL: Clearance
*V*_ss_: Steady-state volume of distribution
*V*_z_: Terminal volume of distribution
*t*_1/2*β*_: Terminal half-life
*t*_1/2,eff_: Effective half-life
POD: Postoperative day.

## Additional Points

*Key Points*. Pharmacokinetic analyses of Gd-EOB-DTPA serum concentrations might have utility as a liver function test. AUC_last_ of Gd-EOB-DTPA showed significant increase between 70% and 90% hepatectomy group. *V*_ss_ showed significant decrease between control and 70% hepatectomy group. Clearance showed negative correlation with an increasing extent of rat hepatectomy.
